# Early identification of and proactive palliative care for patients in general practice, incentive and methods of a randomized controlled trial

**DOI:** 10.1186/1471-2296-12-123

**Published:** 2011-11-03

**Authors:** Bregje Thoonsen, Marieke Groot, Yvonne Engels, Judith Prins, Stans Verhagen, Cilia Galesloot, Chris van Weel, Kris Vissers

**Affiliations:** 1 Department of Anaesthesiology, Pain and Palliative Medicine, Radboud University Nijmegen Medical Centre, P.O. Box 9101, 6500 HB Nijmegen, The Netherlands; 2 Department of Medical Psychology, Radboud University Nijmegen Medical Centre, P.O. Box 9101, 6500 HB Nijmegen, The Netherlands; 3 Comprehensive cancer centre (IKNL), Postbus 1281, 6501 BG Nijmegen, The Netherlands; 4 Department of Family Medicine, Radboud University Nijmegen Medical Centre, P.O. Box 9101, 6500 HB Nijmegen, The Netherlands

## Abstract

**Background:**

According to the Word Health Organization, patients who can benefit from palliative care should be identified earlier to enable proactive palliative care. Up to now, this is not common practice and has hardly been addressed in scientific literature. Still, palliative care is limited to the terminal phase and restricted to patients with cancer. Therefore, we trained general practitioners (GPs) in identifying palliative patients in an earlier phase of their disease trajectory and in delivering structured proactive palliative care. The aim of our study is to determine if this training, in combination with consulting an expert in palliative care regarding each palliative patient's tailored care plan, can improve different aspects of the quality of the remaining life of patients with severe chronic diseases such as chronic obstructive pulmonary disease, congestive heart failure and cancer.

**Methods/Design:**

A two-armed randomized controlled trial was performed. As outcome variables we studied: place of death, number of hospital admissions and number of GP out of hours contacts.

**Discussion:**

We expect that this study will increase the number of identified palliative care patients and improve different aspects of quality of palliative care. This is of importance to improve palliative care for patients with COPD, CHF and cancer and their informal caregivers, and to empower the GP. The study protocol is described and possible strengths and weaknesses and possible consequences have been outlined.

**Trial Registration:**

The Netherlands National Trial Register: NTR2815

## Background

According to the World Health Organization (WHO) palliative care is 'an approach that improves the quality of life of patients and their families facing the problems associated with life-threatening illness, through the prevention and relief of suffering by means of early identification and impeccable assessment and treatment of pain and other problems, physical, psychosocial and spiritual' [1]. A first challenge evoked by this definition is the early identification of a patient who may benefit from palliative care. Although the WHO-recommendations have been accepted worldwide, no scientific papers have been published yet on how to identify patients who could potentially benefit from an earlier start of a palliative care in general practice. A literature review of Qaseem et al. did not identify any validated tools that predict the optimal timing to initiate palliative care services in general practice,[2] despite the fact that a lot of research has been undertaken to elucidate the prediction of mortality, survival, and prognostication for patients with advanced cancer and non-cancer diseases [3-10]. For patients not recognized as being in a palliative phase an individualized well-considered plan of action is missing [11,12].

**Figure 1:**
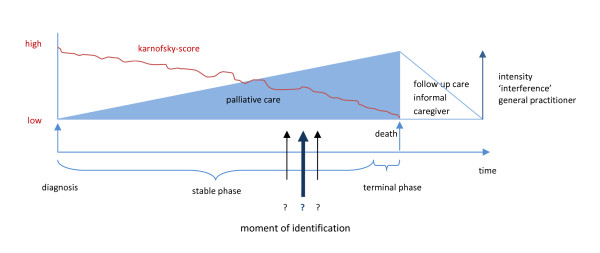
**What is the moment to start palliative care?, a modified figure of Lynn and Adamson**[37] .

Several illness trajectories have been described for people with progressive chronic illnesses [13,14]. For none of these trajectories the right moment to start palliative care has been defined yet. Particularly regarding patients with non-malignant diseases, such as advanced chronic obstructive pulmonary disease (COPD) and congestive heart failure (CHF), recognizing or defining the moment when palliative care should be taken in consideration seems difficult (Figure 1) [15,16]. In a national questionnaire among multiple palliative care providers the lack of prognostic indicators and clinical triggers for starting end of life care appeared to be the most important missing link in applying palliative care in primary care [17]. By integrating palliative care into curative care practices or combining palliative care with disease-oriented management earlier in the disease trajectory, chronically ill patients nearing the end of life reported improved satisfaction with care and demonstrated less acute interventions and were more likely to die at home [18,19].

The second challenge in bringing the WHO definition into clinical practice, for which early identification is a prerequisite, is a structured proactive palliative care planning. Palliative care programs appeared to reduce symptom distress and improve patient and family satisfaction. Important elements of structured proactive palliative care proved to be coaching the patient to make choices regarding future interventions or restrictions,[20-22] consulting caregivers,[18] eliciting values and addressing the psychological, existential and social context of patient and informal caregiver [23,24]. By proactive planning death at home could be enhanced, [25-27] the number of unforeseen transfers decreased,[28] hospital lengths of stay and aggressive interventions diminished and consequently costs and utilization decreased [28-30].

For GPs a structured proactive palliative care planning is a challenge, as patients with an advanced chronic disease are often under supporting care of the disease-specific specialists until far in the disease trajectory [31]. The GPs should pick up their role as coordinator of palliative care against the mainstream of disease-oriented interventions [32,33]. Several studies concluded that when a GP is part of a team, palliative care improves on different aspects; for patient, informal carer and the participating GP [34,35].

### Aims of the study

#### Research questions

In this study we aim to answer the following questions:

Does early identification and proactive palliative care planning of palliative patients by the GP influence

1. Place of death, number of transitions and number of contacts with the out of hours primary care service?

2. Quality of life of patients and their informal caregivers and prescriptions?

3. GP satisfaction with the delivered palliative care and their own assessment of their ability to provide palliative care?

The objective of this report is to present the study protocol used for the data collection in 2009 and 2010. We describe the protocol of the study, provide a description of the intervention, the methodology and the baseline characteristics of the participating GPs. The described methodology will also serve as a reference for future publications about this study.

## Methods/Design

### Study design

We performed a two-armed randomized controlled trial.

We studied the following hypothesis: H_0_: training GPs in early identification of palliative care patients and proactive care planning will not increase the percentage of patients that die at home, will not reduce the amount of hospital admissions and will not reduce the number of contacts with the out of hours primary care service. This hypothesis will be rejected if the training has a significant positive effect on these aspects of care.

### Power calculation

Sample size was based on number of contacts with the out of hours primary care service. To detect a difference between the intervention and the control group with a power of 80% and an alpha error of 0.05 minimum sample size was 96 patients in both groups when 20% reduction in out of hours contacts was considered.

### Participants

GPs in two comprehensive cancer centre (IKO and IKZ) regions in the South-East of the Netherlands were invited by mail to participate in the study. After one month a reminder was send to non-responders. Excluded were GPs who are consultant in palliative care or who are locum. GPs that wanted to participate were stratified for degree of urbanization and working hours (part-time or full-time) and were randomized assigned to the intervention or the control condition by an independent statistician. To prevent contamination, those GPs working together in the same practice were placed in the same study group.

### Ethical considerations

The study was conducted after approval of the research ethics committee of the Radboud University Nijmegen Medical Centre in accordance with the Medical Research Involving Human Subjects Acts (WMO). Patient and physician anonymity was guaranteed throughout the registration and data entry process. Patients and their informal caregiver were invited to participate by their GP. If they agreed to participate in the longitudinal study they received a patient information letter and an informed consent form. Trial registration has been obtained. (The Netherlands National Trial Register: NTR2815)

### The intervention

The intervention for the GPs in the experimental condition consisted of three consecutive parts.

Part one consisted of a five-hours training in early identification of palliative patients and proactive care planning. This training was provided by two experienced GPs with a specialization in palliative care and an extended experience in teaching. Early identification was based on two tools, developed in an earlier stage of the project and described elsewhere (submitted). The first tool is a plasticized card (Table 1) with indicators to identify and recognize patients with respectively COPD, CHF and cancer as being in a stage that palliative care should be considered, the so-called 'Radboud Indicators Palliative Care Needs' (RADPAC). GPs were trained in 1) checking actual problems of the patient in a structured way at the moment of identification, 2) considering potential problems that could be expected in the (near) future and 3) foretelling the most likely scenarios on deterioration and death. A previously developed tool (Table 2) could be used as an abstract of the content of the training and could be used to structure the discussion with the patient and their informal caregiver, and to explore their actual en potential problems and needs (Proactive Palliative Care Planning Card (PPCPC)). The aim was a shared proactive policy to deliver specific, proper and individualized palliative care planning.

**Table 1:** The RADboud indicators of PAlliative Care needs (RADPAC)

Congestive Heart Failure	1. The patient has severe limitations, experiences symptoms even while at rest. Mostly bedbound patients. *(NYHA IV)* 2. There were frequent hospital admissions *(> 3 per year)* 3. The patient has frequent exacerbations of severe heart failure *(> 3 per year)* 4. The patient is moderately disabled; dependent. Requires considerable assistance and frequent care *(Karnofsky-score ≤ 50%)* 5. The patient increases in weight what is not responding to increased dose of diuretics 6. A general deterioration of the clinical situation (oedema, orthopnoe, nycturie, dyspnoea) 7. The patient mentions 'end of life approaching'
**Chronic Obstructive Pulmonary Disease**	1. The patient is moderately disabled; dependent. Requires considerable assistance and frequent care *(Karnofsky-score ≤ 50%)* 2. The patient has substantial weight loss (± 10% loss of bodyweight in six months) 3. The presence of congestive heart failure 4. The patient has orthopnoe 5. The patient mentions 'end of life approaching' 6. There are objective signs of serious dyspnoea (decreased dyspnoea d' effort, dyspnoea with speaking, use of respiratory assistant muscles and orthopnoe)

**Cancer**	1. Patient has a primary tumour with a poor prognosis 2. Patient is moderately disabled; dependent. Requires considerable assistance and frequent care *(Karnofsky-score ≤ 50%)* 3. There is a progressive decline in physical functioning 4. The patient is progressively bedridden 5. The patient has a diminished food intake 6. The presence of progressive weight loss 7. The presence of the anorexie-cachexie syndrome (lack of appetite, general weakness, emaciating, muscular atrophy) 8. The patient has a diminished 'drive to live'

**Table 2:** Reminder for proactive planning and disease specific potential problems

Somatic domain		Social en financial domain	
Policy		Policy	

Actual problems:		Actual problems:	

Expected problems: Scenario of dying:		Expected problems:	


**Care provision and activity of daily living**		**Sense of meaning and psychological domain**	

Policy		Policy	

Actual problems:		Actual problems:	

Expected problems:		Expected problems:	




**Possible future problems** Pain, Dyspnoe, Ileus, Delirium, Fear, Depression, Coma, Liver/renal failure Strain of informal caregiver, Special technical care			

**Disease specific interest** CHF: anaemia switch of the defibrillator weight COPD: medicinal/non-medicinal possibilities against dyspnoea			

(Proactive Palliative Care Planning Card, PPCPC)

The second part of the intervention consisted of a coaching session for the GPs with a physician specialized in palliative care regarding each identified patient included in the study. In this session the GP received feedback and suggestions on the proposed proactive palliative care plan, potential future problems and potential scenarios of deterioration and death.

The third part of the intervention consisted of two peer group sessions of the intervention GPs, eight and ten months after the initial training session. In these sessions the main focus was patient-GP communication techniques regarding having the first conversation with the patient about palliative care (and thus about end of life issues). GPs also had the opportunity to exchange experiences on this topic. Data for process description were collected during the first year after T0. Effect evaluation took place in April 2011.

### Control group

GPs in the control group were asked to provide usual care. They were not trained and had no access to RADPAC, nor to the PPCPC. Consultation by telephone with the palliative care helpdesk of the Comprehensive Cancer Centre was possible as usual. This service is available 24/7 for all GPs in the two comprehensive cancer centre regions in the South-East of the Netherlands; mostly these consultations are on problems in the terminal phase for which the GP needs specialised advice for acute problems. For GPs in the control group a training will be organized after the intervention study is closed.

### Data collection

**Figure 2:**
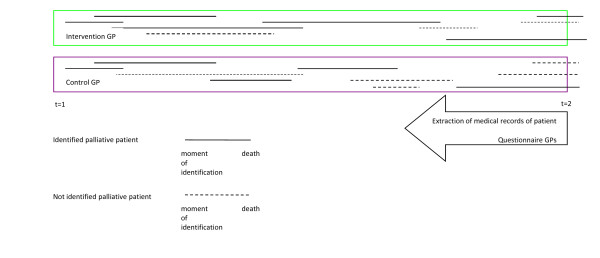
**Study design randomized controlled trial**.

At the start of the study (t = 1), GPs in the intervention group were asked to use RADPAC to screen the medical records of all patients in their practice to identify patients with COPD, CHF or cancer who potentially could benefit from a palliative care approach. They were instructed to continue to use RADPAC each time new data of a patient with CHF, COPD or progressive cancer was available. In 2011, anonymous data were collected retrospectively from the medical records of all patients that had a non-acute death during the intervention period, as well in the intervention as in the control practices (t = 2). All deceased patients who were diagnosed with CHF, COPD and/or cancer were included (Figure 2). Each non-planned (out of hour) contact and hospital or nursing home admission during the study was derived from the medical record and registered as well as place of death and if the patient had been identified as being in the palliative trajectory. Besides, qualitative data were collected concerning proactive care planning in both study groups.

### Outcomes

GPs of both study groups were invited to extract data of all patients that died during the observational period from the medical records: demographics, disease history, place of death, hospital admissions and out of hours consultations. Of each participating GP, demographic characteristics, practice characteristics and their interest in palliative care were collected (Table 3).

**Table 3:** Baseline characteristics participating GPs^# ^(n = 133)

*Characteristics of general practitioners *	
Age - yr	48,2 ± 8,1

Gender male sex - no.(%)	81 (60, 9)

Working week fulltime - no. (%)	70 (52, 6)

Experience - no. (%)	

≤ 1 year	2 (1, 5)

2 - 5 years	14 (10, 5)

6 - 10 years	21 (15, 8)

≥ 10 years	94 (70, 7)

Missing	2 (1, 5)

Interest in palliative care*	8, 14 ± 1, 12

Missing	3

Estimation of own capability**	6, 83 ± 0, 92

Missing	3

*Characteristics of practice *	

Practice form - no. (%)	

Single-handed	28 (21, 1)

Dual	53 (39, 8)

Group and health centres	52 (39, 1)

Missing	0

Degree of urbanisation - no. (%)	

Very	46 (34, 6)

Moderate	28 (21, 1)

Less	41 (30, 8)

No	18 (13, 5)

Missing	0

Size of practice Fte-average practice^+^	1728 ± 409

Missing	1

*Palliative care*	

Palliative patients/y - no. (%)	

≤ 2 patients	10 (7, 5)

3 - 5 patients	72 (54, 1)

5 - 9 patients	43 (32, 3)

≥ 10 patients	6 (4, 5)

Missing	2 (1, 5)

Use of consultant palliative care - no. (%)	

Yes	105 (78, 9)

No	25 (18, 8)

Missing	3 (2, 3)

^#^Plus-minus values are means ± SD

^+ ^Size of practice = fulltime-equivalent/average practice (= 2350 patients)

*Interest in palliative care, visual analogue scale, rang 0, indicating no interest, to 10 very much interest.

** Estimation of own capability, visual analogue scale, rang 0, indicating not capable, to 10 very much capable.

### Data management and plan of statistical analysis

All data were entered in a database and analysed with SPSS 16.0. In both study groups, all patients who died during the inclusion and observation period of the study of a non-acute death, were included in the retrospective analysis. Intervention and control group will be compared on the main outcomes (place of death, number of transitions and out of hours contact, amount of identified patients). Recruitment rates and drop-out rates will be calculated. In case denominators prove to be significantly correlated with the outcome of the main study question, an Anova will be performed to identify potential related or independent factors. Multilevel analysis will be performed.

## Discussion

The present study has been designed to assess the effects of training GPs in early identification and a proactive palliative care approach regarding patients with COPD, CHF or cancer.

### Strengths

Up to now, hardly any data are available about implementing the 2002 WHO-definition for palliative care. To our knowledge this is the first intention to treat RCT that assesses the effect of training GPs in early identification and using a proactive holistic palliative care approach, which are the main aspects of this definition. The training for the GPs in the intervention group was standardised and piloted, to minimize differences between the two trainers and to be available for future courses. The tools that the GPs in the intervention group could use for helping to identify palliative patients in an earlier stage than usual and to structure the proactive care planning, were developed in a scientifically sound way. Results will be published in peer-reviewed scientific journals and will be communicated to relevant clinician associations.

### Weaknesses

Those GPs that were interested to take part in the study, probably have a special interest in palliative care. Besides, as there is a lot of attention for proactive palliative care in medical journals and in Dutch policy, GPs in the control group might be influenced by new information or followed courses. This implies that it might be difficult to find significant differences between intervention and control group. As patients were identified by their own GP, we were not able to influence this process directly.

We did not choose to collect prospective patient data in the control group, as this would be a sort of intervention. Therefore, effect measurement took place retrospectively. GPs in both intervention and control group were asked to collect retrospective data from their digital patient information system. This implies that we do not have patient data of non-responding GPs.

We performed a multifaceted intervention: a combination of training GPs and offering them tools to facilitate early identification and proactive care planning. Usually, multifaceted interventions are more effective than single interventions,[36] but the relative impact of each component of the intervention cannot be established.

## Conclusion

The present study will increase the knowledge about the effect of training GPs in early identification and a proactive palliative care approach. This knowledge is of importance to improve palliative care for patients with COPD, CHF and cancer and their informal caregivers, as well as to empower the GP. Here, the study protocol is described and possible strengths and weaknesses and possible consequences have been outlined.

## Competing interests

The authors declare that they have no competing interests that are directly relevant to the content of this article.

## Authors' contributions

BT led the drafting of this paper and together with MG, SV and YE the development of the protocol. YE and KV were the initiators of the study and obtained funding. YE and MG did study supervision. YE, MG, SV, CW and KV were responsible for critical revision of the manuscript. All authors read, revised and approved the final manuscript.

## Pre-publication history

The pre-publication history for this paper can be accessed here:

http://www.biomedcentral.com/1471-2296/12/123/prepub
